# A randomised, controlled trial of a dietary intervention for adults with major depression (the “SMILES” trial): study protocol

**DOI:** 10.1186/1471-244X-13-114

**Published:** 2013-04-15

**Authors:** Adrienne O’Neil, Michael Berk, Catherine Itsiopoulos, David Castle, Rachelle Opie, Josephine Pizzinga, Laima Brazionis, Allison Hodge, Cathrine Mihalopoulos, Mary Lou Chatterton, Olivia M Dean, Felice N Jacka

**Affiliations:** 1 IMPACT Strategic Research Centre, Deakin University, Deakin, VIC, Australia; 2 School of Public Health and Preventive Medicine, Monash University, Monash, VIC, Australia; 3 ORYGEN Research Centre, University of Melbourne, Melbourne, VIC, Australia; 4 Department of Psychiatry, University of Melbourne, Melbourne, VIC, Australia; 5 Department of Dietetics, Faculty of Health Sciences, Latrobe University, Latrobe, VIC, Australia; 6 Department of Psychiatry, St Vincent’s Hospital, Melbourne, VIC, Australia; 7 Department of Medicine, The University of Melbourne, Melbourne, VIC, Australia; 8 Cancer Epidemiology Centre, The Cancer Council Victoria, Melbourne, VIC, Australia; 9 Population Health Strategic Research Centre, Faculty of Health, Deakin University, Deakin, VIC, Australia; 10 Florey Institute of Neuroscience and Mental Health, Parkville, VIC, Australia

**Keywords:** Diet, Nutrition, Depression, Mental health, Social support

## Abstract

**Background:**

Despite increased investment in its recognition and treatment, depression remains a substantial health and economic burden worldwide. Current treatment strategies generally focus on biological and psychological pathways, largely neglecting the role of lifestyle. There is emerging evidence to suggest that diet and nutrition play an important role in the risk, and the genesis, of depression. However, there are limited data regarding the therapeutic impact of dietary changes on existing mental illness. Using a randomised controlled trial design, we aim to investigate the efficacy and cost-efficacy of a dietary program for the treatment of Major Depressive Episodes (MDE).

**Methods/Design:**

One hundred and seventy six eligible participants suffering from current MDE are being randomised into a dietary intervention group or a social support group. Depression status is assessed using the Montgomery–Åsberg Depression Rating Scale (MADRS) and Structured Clinical Interview for Diagnostic and Statistical Manual of Mental Disorders (Non Patient Edition) (SCID-I/NP). The intervention consists of 7 individual nutrition consulting sessions (of approximately 60 minutes), delivered by an Accredited Practising Dietitian (APD). Sessions commence within one week of baseline assessment. The intervention focuses on advocating a healthy diet based on the Australian Dietary Guidelines and the Dietary Guidelines for Adults in Greece. The control condition comprises a befriending protocol using the same visit schedule and length as the diet intervention. The study is being conducted at two locations in Victoria, Australia (a metropolitan and regional centre). Data collection occurs at baseline (pre-intervention), 3-months (post-intervention) and 6– months. The primary endpoint is MADRS scores at 3 months. A cost consequences analysis will determine the economic value of the intervention.

**Discussion:**

If efficacious, this program could provide an alternative or adjunct treatment strategy for the management of this highly prevalent mental disorder; the benefits of which could extend to the management of common co-morbidities including cardiovascular disease (CVD), obesity, and type 2 diabetes.

**Trial registration:**

NCT01523561

## Background

Depression is currently a leading cause of health and economic burden, internationally [1]. Increased investment in recognition and treatment has failed to improve depression outcomes substantially in recent years, suggesting that other factors may be influencing the burden of this condition [2]. Traditionally, treatment of depression has primarily focused on targeting biological and psychological pathways [3,4]. Accumulating evidence now suggests that lifestyle factors such as diet quality contribute to a number of mental illnesses [5] and play an important role in the risk and genesis of depression specifically [6]. Dietary modification is widely recognised and promoted for the primary prevention of non-communicable disorders, such as cardiovascular disease (CVD), obesity and diabetes, yet has not been considered for the management of mental illness. To date, there are virtually no data regarding the therapeutic impact of dietary changes on depression.

This impact of habitual diet on the common mental disorders, is garnering significant scientific and public interest worldwide. There are many published studies providing consistent support for an association between habitual diet quality and depression [7-12]. For example, in a population based sample of 1046 Australian women aged 20–94 years, a ‘healthy’ dietary pattern was associated with a reduced likelihood of clinically diagnosed depressive disorders, whereas a dietary pattern comprising processed and ‘unhealthy’ foods was associated with an increased likelihood of psychological symptoms and depression [7]. Higher diet quality scores were also associated with reduced psychological symptoms [7]. The associations between diet quality and mental illness have also been shown in the Hordaland Health Study of 5731 adults in Norway where participants with better quality diets were less likely to be depressed or anxious [8]. This association was further investigated in a cross sectional study of more than 7000 Australian adolescents that found dose–response relationships between two measures of diet quality: healthy (negative) and unhealthy (positive) and the likelihood of adolescent depression, after adjustment for confounders [9]. A population based study has also demonstrated a cross sectional and longitudinal relationship between diet quality and mental health in approximately 3000 Australian adolescents [10]. Importantly, improvements in diet quality were mirrored by improvements in mental health, while reductions in diet quality were associated with declining psychological functioning over the two year follow up period [10].

Similarly, recent prospective data from the SUN Cohort study in Spain demonstrated an inverse association between the level of adherence to a Mediterranean dietary pattern and the risk for incident depression in more than 10,000 adults [11]. The Whitehall II cohort study of 3486 participants found an increased risk of self-reported depression after five years for those adhering more strongly to a ‘western’ style diet pattern, and a reduced risk for those following a ‘whole foods’ diet pattern [12]. Interestingly, these studies have identified that when ‘unhealthy’ dietary patterns were assessed in relation to depression, they showed positive associations with depression [7,9,10,12], indicating that what is excluded from the diet may be as important as what is included.

These recent data consistently support a role for diet quality in depressive illness [7-12]. However, there are currently no available data regarding the therapeutic impact of dietary changes on existing mental illness. Studies involving dietary interventions, in non-psychiatric conditions, have demonstrated that diet can be successfully modified, leading to physiological changes that have implications for improved mental health [13,14]. Taken together with previous data indicating success with individualised nutrition education counselling in those with mental illness [15,16], an intervention targeting dietary improvement may also be feasible for individuals with depression. If found efficacious, such a treatment strategy has the advantage of being of population wide public health importance, and of substantial additional benefit across many disease states [6].

This paper presents the study protocol for the SMILES trial: “Supporting the Modification of lifestyle In Lowered Emotional States.” This is a randomised controlled trial that aims to investigate the efficacy and cost-efficacy of dietary improvement in the treatment of Major Depressive Episodes (MDE). We hypothesise that a structured dietary intervention, focusing on dietary improvement, will be superior to a control condition (befriending) in the treatment of MDE.

## Methods

### Study design

This is a 12-week, parallel group, single blind, randomised, controlled trial of a dietary intervention for the treatment of moderate to severe depression. We are currently enrolling 176 participants over two sites; Barwon Health in Geelong and St. Vincent’s Health in Melbourne (Victoria, Australia). Recruitment and intervention are anticipated to occur over a 1.5-2 year period. Participants are randomised to receive either the dietary intervention or a control condition (social support). Participants in both groups complete assessments prior to program commencement (baseline), at program completion (3-months) and at a 6-month follow-up (via telephone).

### Study aims

We aim to investigate the efficacy of dietary improvement in the treatment of MDE. The primary outcome includes changes in MDE using the Montgomery-Åsberg Depression Rating Scale (MADRS). Secondary outcomes include; depressive and anxiety symptoms, functioning, quality of life, and changes in targeted dietary behaviours, cardiovascular and metabolic risk. A secondary aim is to evaluate the cost efficacy of the intervention from a societal perspective at 3 months.

### Participant eligibility

#### Inclusion criteria

Eligibility criteria includes participants who are: aged 18 or over and can provide informed consent; successfully fulfill the Diagnostic and Statistical Manual of Mental Disorders 4 (DSM-IV-TR) diagnostic criteria for Major Depressive Disorder, Single Episode or Recurrent, score 18 or over on the MADRS [17] and score 68 or less in a Dietary Screening Tool (DST) [18], modified for Australian diets, during screening. If participants are on antidepressant therapy, they are required to be on the same treatment for at least two weeks prior to randomisation. Participants must be readily available for a 3-month period and have the ability to eat foods as prescribed, without religious, medical, socio-cultural or political factors precluding participation or adherence to the diet.

#### Exclusion criteria

Participants are ineligible if they have: a concurrent diagnosis of bipolar I or II disorder; two or more failed trials of antidepressant therapy for the current MDE; known or suspected clinically unstable systemic medical disorder; pregnancy; commencement of new psychotherapy or pharmacotherapy within the preceding fortnight; severe food allergies, intolerances or aversions; current participation in an intervention targeting diet or exercise; a primary clinical diagnosis of a personality disorder; or a current substance use disorder.

### Sample recruitment

Community based recruitment strategies are used to recruit study participants. These include advertisements in local newspapers and radio stations; flyers in medical waiting rooms, pharmacies and university campuses; newsletters; and contact with potential referral sources, for example, general practitioners, private psychiatrists and local psychiatric inpatient units. Social media, such as Twitter and Facebook, is also used as a means of recruitment.

Prospective participants express their interest to the Clinical Trial Co-ordinator (CTC) through email or a phone call. Trained Research Assistants (RA) assess initial eligibility using a specifically developed telephone screening tool. If the individual meets these preliminary inclusion criteria, they are invited to attend a face-to-face screening session to determine full eligibility.

### Study procedure

#### Screening assessment

Prospective participants complete the DST [18] to confirm “poor” dietary quality before enrolment. In order to adequately test the hypothesis that improving diet quality will reduce depressive symptomatology, those who have limited scope for improving their diet (DST scores ≥69) are excluded. If their score meets eligibility, the MADRS is administered to determine current depression severity. Depression status is subsequently confirmed using a psychiatric diagnostic interview (Structured Clinical Interview for DSM-IV, Non-patient Edition; SCID-I/NP). The Standardised Assessment of Personality – Abbreviated Scale (SAPAS) [19] is used to screen for personality disorder. Once deemed to be eligible, participants complete a 7 day food diary and a food frequency questionnaire (FFQ) in the week leading up to baseline assessment. Participants attend a local (nominated) pathology clinic to provide fasting blood samples, before undertaking baseline assessment and randomisation (using a computer generated randomisation table) to either study condition.

### Study conditions

#### Diet intervention group

The intervention is a Modified Mediterranean diet, based on the Australian Dietary guidelines [20] and the Dietary Guidelines for Adults in Greece [21]. The diet is rich in vegetables, fruit and whole grains with an emphasis on increased consumption of oily fish, olive oil, legumes and raw unsalted nuts. Moderate consumption of lean read meat and reduced fat dairy is included. The diet comprises the following energy ratios: 39% total fat (e.g. 22% monounsaturated fat), 37% carbohydrates, 17% protein. In order to tailor the diet to a mental health population, the diet was designed to be manageable, sustainable and easy to follow. The primary focus is on increasing diet quality while reducing intake of energy dense, nutrient poor foods.

Participants receive seven individual sessions of approximately 60 minutes each, delivered by an APD. The first four sessions occur weekly and the remaining three sessions occur fortnightly. At the first session the dietitian conducts a diet history (incorporating a check list of commonly consumed foods) to assess usual dietary intake. Food models and metric measuring utensils are used to assist with the estimation of portion sizes.

Participants are provided with supporting written information specifically designed for the intervention to assist with achieving dietary adherence. These resources include healthy eating guidelines, convenient meal ideas, shopping lists, meal plans and recipe ideas. To encourage dietary adherence (and display the variety of foods that form the diet), participants are provided with a food hamper, incorporating the main components of the Modified Mediterranean diet. Recipes and meal plans using foods in the hamper are also available.

Additional sessions may incorporate label reading, food security (access to healthy food), motivational interviewing techniques and mindful eating as part of the individualised dietetic service. Participants are encouraged to set personalised goals at each session and identify barriers/motivators to change. At the final session, goals achieved are summarized with longer term strategies for achieving sustainable change discussed. The hamper is also provided at this session to promote longer term adherence.

#### Social support group

The control condition comprises a befriending protocol [22], using the same visit schedule and length as the diet intervention. Befriending consists of a trained personal talking about neutral topics of interest to the participant with the intention of keeping the participant engaged and positive. The befriending condition [22] aims to control for four factors: time, client expectancy, therapeutic alliance, and therapist factors when comparing to the intervention group in a RCT. This is done without engaging in techniques specifically used in the major models of psychotherapy. It is often used as a controlled condition for clinical trials of psychotherapy [22]. Befriending is a highly effective validated controlled condition due to the simplicity of the program, and evidently appropriate to participants suffering from mental illness [22].

In the penultimate session, both the dietitian and befriender provide each participant; pathology kits, 7-day Food Diary, Cancer Council of Victoria FFQ (CCVFFQ) [23] and International Physical Activity Questionnaire (IPAQ) [24] to be completed prior to the final session. Participants in the control group are provided movie tickets as compensation for their time and participation in the study.

### Data collection and outcome measures

Table 1 displays the outcome measures and corresponding timepoints. Demographic data (including age, sex, post code, contact details, nominated General Practitioner (GP)), psychiatric, and self-report measures are obtained from each participant at baseline and post intervention (3-months) to determine depressive and anxiety symptoms, functioning and quality of life (using the Assessment of Quality of Life 8 dimension [AQoL8D] [25]) which will also allow the calculation of quality-adjusted life-years (QALYs). Clinical data such as blood pressure and anthropometric measures including height, weight and waist circumference are also taken. Habitual dietary patterns are collected as stated above. The IPAQ [24] and self-report smoking questionnaire are completed. At 6-months, participants are contacted via telephone during which time RAs re-administer the MADRS, Hospital Anxiety and Depression Scale (HADS) [26] and DST. At this time, participants initially assigned to the control condition are offered the resources developed and used by the APD for the diet intervention.

**Table 1:** Schedule of measurements

**Variable**	**Instrument**	**Time point**
**Self-report**		
Diet quality	Diet screening tool [18]	Screening, 6-months^#^
Psychiatric conditions	SCID-I/NP*	Screening
Personality disorders	Standardised Assessment of Personality – Abbreviated Scale (SAPAS) [19]	Screening
Depression	MADRS* [17]	Screening, baseline, 3-, 6-months^#^
Anxiety	Hospital anxiety depression scale [26]	Baseline, 3-, 6-months
Depression severity	Clinical global impressions scale [27]	3-months
Mood	Profiles of Moods State (POMS) [28]	Baseline, 3-months
Quality of life, utility, wellbeing	Assessment of Quality of Life (AQoL-8D) [25]; WHO wellbeing scale [29]	Baseline, 3-months
Diet intake	Food Frequency Questionnaire (FFQ) [23]	Baseline, 3-months
Physical activity	International Physical Activity Questionnaire (IPAQ) [24]	Baseline, 3-months
Smoking	Self-report	Baseline, 3-months
Medications	Self-report	Baseline, 3-months
Medical conditions (including family history)	Self-report (Doctor diagnosed) depression, anxiety, myocardial infarction, angina, hypercholesterolemia, high blood pressure, diabetes	Baseline, 3-months
Health care resource utilisation	Self-reported hospitalisations, attendances at physicians, psychologists, allied health, complementary and alternative medicine providers	Baseline, 3-months
Self efficacy	General Self Efficacy Scale (GSE) [30]	Baseline, 3-months
Participant satisfaction	Diet preference score	3-months
Lost productivity	Self-reported time lost from paid and unpaid work	Baseline, 3 months
**Anthropometric & clinical**		
Waist, weight, height, blood pressure	Height to nearest 0.1 cm. Body weight to nearest 0.1 kg. BMI = weight divided by height squared kg/m^2^, Digital metre	Baseline, 3-months
**Biochemical**		
Erythrocyte fatty acids and cartenoids	Venous blood sample (fasting)	Baseline, 3-months
High-density lipoprotein, Lower-density lipoprotein, total cholesterol	As above	Baseline, 3-months
Triglycerides, blood glucose	As above	Baseline, 3-months

* clinician administered; ^#^6-month assessment via telephone. Baseline= pre-intervention, 3-months= post-intervention, 6-months= 3-months post-intervention.

Health care resource utilization is captured through self-report and includes information on the quantity and reasons for hospitalisations, numbers of visits to health care providers and complementary and alternative medicine practitioners as well as any out-of-pocket costs related to services for both mental and physical health. Previous studies have found associations between MDE and high total resource utilization and costs (both mental and physical disorders) [31,32]. Randomisation should distribute underlying characteristics that may confound between-group comparisons and will be evaluated by analysis of baseline data. Lost productivity is similarly self-reported using questions to capture time away from paid and unpaid work due to health problems (absenteeism), in addition to time working while suffering from health problems (presenteeism).

Blood samples are prepared by the nominated laboratory as per the standardised protocol. Once prepared, samples are temporarily stored in −20 degree freezers and then transferred to −80 degree freezers at the Department of Dietetics and Human Nutrition, La Trobe University, Bundoora and Barwon Health, Geelong until assays are performed in batches. Erythrocyte fatty acids and plasma carotenoids represent important markers of dietary intake/change in both groups. Erythrocyte fatty acids will be analysed at the Metabolic Research Unit, Deakin University. Retinol, alpha- and gamma-tocopherol and carotenoids (lutein + zeaxanthin, beta-cryptoxanthin, lycopene and alpha- and beta-carotene) in plasma will be processed and analysed by High Pressure Liquid Chromatography at La Trobe University.

Dietary intake is assessed for each participant at baseline and post intervention to evaluate intake relative to the Australian Dietary Guidelines. Detailed information on foods and fluids consumed are collected using 7-day food diaries at the beginning and end of the intervention (1–7 days preceding the first session and 1–7 days preceding the final session) to monitor adherence to the intervention. These dietary assessments are also administered to the control group to capture any dietary changes made while being involved in the study.

RAs conducting data collection are blinded to participants’ group allocation. Prior to each assessment, participants are reminded not to reveal the group to which they have been assigned.

### Data management

The RA at respective sites are responsible for electronic scanning and storage of hard copies of case report forms (CRFs) for entry into the password protected central database by the respective RAs. Hard copies are kept in a secure filing cabinet at the respective sites. If requested, participants can access their individual results at the completion of the study period, by making a direct request to the CTC.

### Study integrity

Approval to conduct the study was received from Human Research Ethics Committees of St. Vincent’s Hospital Barwon Health and Deakin University. Written informed consent is obtained from all participants. This study has been developed in accordance with CONSORT guidelines. Safety protocols have been developed at each site to monitor the welfare of participants whose condition may deteriorate throughout the course of the study, with senior psychiatric consultants (DC, MB) overseeing this process.

### Sample size

We are aiming to recruitment 88 participants per group, assuming attrition of 15% over 6-months (leaving 70 individuals per group eligible for evaluation), with 8 predictors (including potential confounding variables). For a one- tailed analysis with α=0.05, the study will be powered at 80% to detect a true difference in rating scale score between the diet and befriending groups if the effect size is 0.15 or greater.

### Data analyses

Data analysis will be conducted by a researcher blinded to the treatment conditions. A one-tailed analysis will be used to detect differences in MADRS scores between the intervention and control groups. Analyses will explore dose–response effects associated with intervention diet adherence. The primary efficacy analysis will assess average treatment group differences for the primary outcome measure (MADRS) over the entire study period and use a likelihood based mixed-effects model, repeated measures (MMRM) approach at each time point. Analysis of covariance (ANCOVA) will be used to compare differences between treatment means in changes from baseline to follow up. Intention to treat analysis will be employed. To assess confounding, covariates will include age, gender, Body Mass Index (BMI) physical activity levels, smoking and alcohol consumption. Non-parametric statistics will be used when assumptions for parametric methods are violated. Effect sizes will be calculated using Cohen’s guidelines. All tests of treatment effects will be conducted using an alpha level of 0.05 and reporting 95% confidence intervals. Multiple imputation will be employed for the management of missing data.

The economic evaluation will compare the dietary intervention to its comparator (the befriending group) in terms of both the costs and consequences as assessed by an incremental cost-effectiveness ratio (ICER). The primary study design is that of a cost consequence analysis (CCA) [33].

Standard Australian unit costs (i.e. Medicare Benefits Schedule) will be applied to the resource use units collected. Costs will be presented in total and disaggregate forms such as those falling upon the health sector, patients, government and other sectors. The technique of ‘bootstrapping’ will be used to obtain confidence intervals for cost effectiveness ratios, since parametric techniques are inappropriate for use on skewed variables and ratios. The sensitivity of the results will also be tested against the variation in the utility weights and unit cost prices.

## Discussion

It is well-accepted that significant numbers of people with depression fail to respond to pharmacological and/or psychological treatments. It has been suggested that lifestyle components, including diet quality, play a role in the genesis of depression and that lifestyle is an important, yet neglected, intervention target for the treatment of MDE. However, there have been no comprehensive studies to date that have specifically sought to answer the question “if I improve my diet will I feel less depressed?” What is an increasingly common question, both in clinical practice and the general community, remains unanswered to date and this is an important gap in our knowledge base. Our study will provide essential evidence regarding the efficacy and cost-efficacy of dietary improvement in the treatment of depressive illness, thus allowing for the development of treatment strategies incorporating dietary improvements. Addressing lifestyle factors may additionally contribute to secondary prevention, as well as overlapping heavily with validated strategies for the management of other highly prevalent medical conditions such as CVD, obesity and type 2 diabetes. These are conditions associated with depressive illness, and may share common underlying pathways. A dietary intervention for depression as an adjunct to standard care, has the potential to be cost-effective, highly acceptable and widely applicable. This approach may lead to improved outcomes for individuals with MDE and reduce the public health burden of psychiatric illness.

## Abbreviations

CVD: Cardiovascular disease; MDE: Major Depressive Episodes; MADRS: Montgomery–Åsberg Depression Rating Scale; DSM-IV-TR: Diagnostic and Statistical Manual of Mental Disorders 4; SCID-I/NP: Structured Clinical Interview for Diagnostic and Statistical Manual of Mental Disorders (Non Patient Edition); DST: Dietary Screening Tool; SAPAS: Standardised Assessment of Personality – Abbreviated Scale; FFQ: Food frequency questionnaire; CCVFFQ: Cancer council victoria food frequency questionnaire; APD: Accredited Practising Dietitian; CTC: Clinical Trial Co-ordinator; RA: Research Assistant; IPAQ: International Physical Activity Questionnaire; GP: General practitioner; AQoL8D: Assessment of quality of life – 8 dimension; QALYs: Quality-adjusted life-years; CRFs: Case report forms; MMRM: Mixed-effects model, repeated measures; ANCOVA: Analysis of covariance; BMI: Body Mass Index; ICER: Incremental cost-effectiveness ratio; CCA: Cost consequence analysis; CUA: Cost-utility analysis

## Competing interests

MB has received Grant/Research Support from the NIH, Simons Foundation, CRC for Mental Health, Stanley Medical Research Institute, MBF, NHMRC, Beyond Blue, Geelong Medical Research Foundation, Bristol Myers Squibb, Eli Lilly, Glaxo SmithKline, Organon, Novartis, Mayne Pharma, Servier and Astra Zeneca. He has been a paid consultant for Astra Zeneca, Bristol Myers Squibb, Eli Lilly, Glaxo SmithKline, Janssen Cilag, Lundbeck and Pfizer and a paid speaker for Astra Zeneca, Bristol Myers Squibb, Eli Lilly, Glaxo SmithKline, Janssen Cilag, Lundbeck, Organon, Pfizer, Sanofi Synthelabo, Solvay and Wyeth. OMD has received grant support from the Brain and Behavior Foundation, Simons Autism Foundation, Stanley Medical Research Institute, Lilly, NHMRC and an ASBD/Servier grant. FNJ has received Grant/Research support from the Brain and Behaviour Research Institute, the National Health and Medical Research Council, Australian Rotary Health, the Geelong Medical Research Foundation, the Ian Potter Foundation, Eli Lilly and The University of Melbourne and has been a paid speaker for Sanofi-Synthelabo, Janssen Cilag and Eli Lilly.

## Authors’ contributions

AO and FNJ initiated and coordinated the study and finalised the study protocol. AO and JP developed the first draft of the manuscript and subsequent versions. FNJ, MB, DC, LB CI, AH, CM conceptualised and designed the study. OMD and MLC assisted with the development of the study. RO, JP and CI developed the dietary intervention. All authors contributed to drafts of the manuscript and approved the final version.

## Pre-publication history

The pre-publication history for this paper can be accessed here:

http://www.biomedcentral.com/1471-244X/13/114/prepub
